# An Adult Zebrafish Diet Contaminated with Chromium Reduces the Viability of Progeny

**DOI:** 10.1089/zeb.2017.1514

**Published:** 2018-04-01

**Authors:** Marc T. Tye, Jacob E. Montgomery, Maurine R. Hobbs, Kayce T. Vanpelt, Mark A. Masino

**Affiliations:** ^1^ Zebrafish Core Facility, University of Minnesota Twin-Cities, Minneapolis, Minnesota.; ^2^ Department of Neuroscience, University of Minnesota Twin-Cities, Minneapolis, Minnesota.; ^3^ Centralized Zebrafish Animal Resource, University of Utah, Salt Lake City, Utah.

**Keywords:** chromium, brine shrimp, diet, arsenic, barium, zebrafish

## Abstract

The lack of standardized diet for laboratory animals can have profound effects on animal health and lead to less reproducible research outcomes. Live diets are commonly used in zebrafish culture and, although they are a more natural feed than flake or pellet food, are also a potential source of pathogens and toxic compounds. Heavy metals are a group of such compounds, which can accumulate in fish leading to developmental abnormalities, reduced growth, and increased rates of mortality. Two to three weeks after feeding adult zebrafish a new lot of nonhatching decapsulated brine shrimp cysts (Decaps), embryos at the University of Minnesota Zebrafish Core Facility (ZCF) and the University of Utah Centralized Zebrafish Animal Resource (CZAR) began to exhibit an orange color in the yolk, and larval health began to decline. The concentration of chromium in the Decaps (69.6 mg/kg) was more than 30 times that of other zebrafish diets tested (up to 2.1 mg/kg) and is thought to be the cause of the observed symptoms. Within 3 weeks of removing the Decaps from the feeding regimen, the orange coloration in the yolks began to diminish, the morphological abnormalities began to subside, and larval survival rates began to increase. Thus, implementation of standardized zebrafish diets and regular feed-quality testing may help to prevent the introduction of contaminants to zebrafish research facilities.

## Introduction

Many aspects of zebrafish husbandry, including diet, are yet to be standardized. Feeding protocols vary widely between facilities and can include a combination of live feed and/or commercially formulated fish food.^1^ Brine shrimp (*Artemia salina*) are an excellent source of nutrition for zebrafish^2^ and are typically purchased as cysts because they are hardy and can be stored for long periods of time. The cysts are soaked in a brine solution causing them to hatch, which removes the indigestible chorion, and the resulting nauplii are fed to adult zebrafish. Nonhatching decapsulated brine shrimp cysts (Decaps) are a commercially available product in which the chorions are chemically removed and, thus, can be fed directly to fish without the need for hatching.^3^

Diets that include natural foods, such as brine shrimp, are potential routes for pathogens^4,5^ and toxic compounds^6^ to enter a zebrafish facility. The presence of these contaminants can lead to reduced fecundity, poor animal health, and increased rates of mortality, ultimately impacting research outcomes and reproducibility. Commercial pellet and flake foods can also be a source of unwanted compounds such as isoflavones, antinutritional factors, and dyes.^7^ Testing feed for such pathogens and compounds is an uncommon practice in most zebrafish facilities due to its high cost, lack of data pertaining to what is satisfactory for zebrafish husbandry, and the assumption that the feed is free of these contaminants.

Heavy metals, an example of such hazardous compounds, accumulate in fish tissue through food consumption or uptake from surrounding water.^8^ Once in the body, heavy metals have been demonstrated to alter enzyme synthesis and activity, act as endocrine disruptors, and cause osmotic disturbances in developing fish.^9^ Exposure to heavy metals can ultimately result in developmental retardation, disruption of metabolic processes, morphological abnormalities, and death.^9^ This report describes the pathological symptoms that were observed in zebrafish embryos and larvae at the University of Minnesota Zebrafish Core Facility (ZCF) and the University of Utah Centralized Zebrafish Animal Resource (CZAR), the steps that were taken to identify chromium as a contaminant introduced through food, and the steps that were taken to remedy the problem.

## Materials and Methods

### Animal care

Adult and juvenile zebrafish were maintained at the University of Minnesota ZCF and the University of Utah CZAR with a photoperiod of 14-h light/10-h dark cycle. Recirculating water system parameters recorded during the course of the study are described in Table 1. Embryos and larvae were raised in Petri dishes containing 60 μg/mL Instant Ocean (Blacksburg, VA) Sea Salt in 28.5°C incubators. For raising embryos/larvae in lighted conditions, an incubator with a fluorescent lamp on a 14-h light/10-h dark cycle was used. Embryos/larvae raised in dark conditions were either placed in an opaque box inside of a lighted incubator or in an unlit incubator with a tinted glass door. All animal care protocols were approved by the University of Minnesota and University of Utah Institutional Animal Care and Use Committees.

**Table 1.** Recirculating System Water Conditions

	*ZCF*	*CZAR*
Temperature	27.4–29.5°C	27.4–29.5°C
Ammonia	0 ppm	0 ppm
Nitrate	<60 ppm	<30 ppm
Nitrite	<0.5 ppm	<0.5 ppm
Hardness	75–200 ppm	120 ppm
Chlorine	0 ppm	0 ppm
Alkalinity	0–80 ppm	—
pH	7.2–7.6	7.2–7.6
Conductivity	700–750 μS/cm	500–650 μS/cm

ZCF, zebrafish core facility; CZAR, centralized zebrafish animal resource.

### Hatching brine shrimp nauplii

Hatched brine shrimp nauplii were harvested 24 h after seeding 60 g of cysts into 17 L of 33 ppm sodium chloride and grown at 28°C overnight with aeration. Unhatched cysts and shells were separated from hatched nauplii by collecting differentially settled fractions from the cone. Nauplii were rinsed thrice under running reverse osmosis (RO) water before shipping for analysis.

### Imaging

Images of unanesthetized embryos and larvae were collected with a Sony (Tokyo, Japan) DCR-SR300 digital video camera and an AmScope (Irvine, CA) MU1000 camera mounted to a Leica Microsystems (Wetzlar, Germany) MZ9.5 stereomicroscope. Brightness and contrast adjustments were made to entire panels using Photoshop CS5 (Adobe Systems, San Jose, CA).

### Carotenoid testing

Two lots of nonhatching decapsulated brine shrimp cysts (Decaps; Brine Shrimp Direct, Ogden, UT) were submitted to the University of Missouri Experiment Station Chemical Laboratories (Columbia, MO) from the ZCF for carotenoid testing. Total carotenoids were tested using colorimetric method AOAC Official Method 938.04.

### Heavy metal testing

All feeds tested by the ZCF were submitted to Pace Analytical Services, LLC (Minneapolis, MN) for heavy metal analysis. The CZAR submitted Decaps from two suppliers (Brine Shrimp Direct and Your Fish Stuff, Lebanon, NJ) to Pace Analytical Services, LLC. Heavy metal analysis of feeds at Pace Analytical Services, LLC was completed using inductively coupled plasma-mass spectrometry (EPA Method 6020). The CZAR also submitted unhatched brine shrimp cysts (INVE, Salt Lake City, UT) and frozen 24 h-old (first instar stage) nauplii hatched from the cysts to Analytical Laboratories (Boise, ID). Heavy metal testing at Analytical Laboratories was conducted using inductively coupled plasma-atomic emission spectrometry (U.S. EPA Method 200.7). Embryos and water samples from the ZCF were sent to Pace Analytical Services, LLC, where they were tested for heavy metals using inductively coupled plasma-mass spectrometry (EPA Methods 6020 and 200.8, respectively). Appropriate controls and adjusted method detection limits (MDLs) were provided for each sample by the testing laboratories. Heavy metal concentrations in all feed and tissue samples tested above their respective MDL unless specified otherwise (indicated as <MDL, with MDL for the sample provided).

## Case Report

### Symptoms

**FIG. 1.**
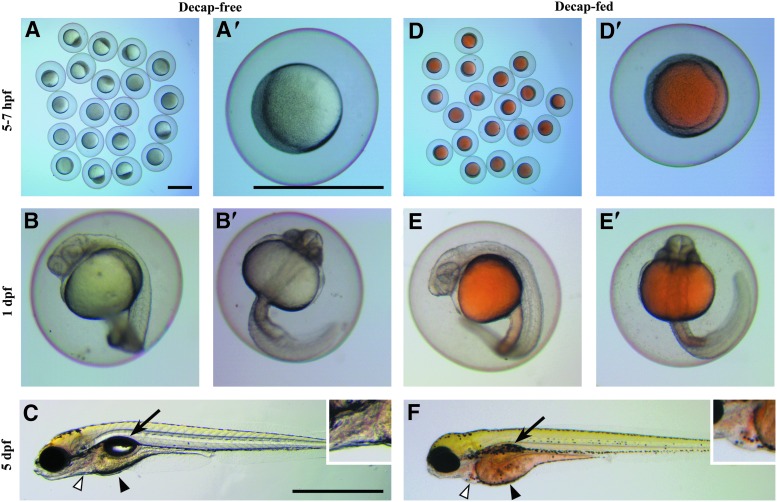
Comparison of yolk coloration and morphological deformities between progeny of adults that were fed decapsulated brine shrimp cysts (Decap-fed) and progeny of adults that were not fed decapsulated brine shrimp cysts (Decap-free). **(A–C)** Progeny of Decap-free adults exhibited clear or pale *yellow* yolks and normal morphological features. Low **(A)** and high **(A′)** magnification of 5–7 hpf embryos. Lateral **(B)** and ventral **(B′)** views of 1 dpf embryos. **(C)** Lateral view of a 5 dpf larva with normal inflated swim bladder (*arrow*), cardiac sac (*white arrowhead* and *inset*, enlarged 200%), and pale-*yellow* yolk (*black arrowhead*). **(D–F)** Progeny of Decap-fed adults exhibited *orange* coloration in yolks and abnormal morphological features. Low **(D)** and high **(D′)** magnification of 5–7 hpf embryos with *orange*-*colored* yolks. Lateral **(E)** and ventral **(E′)** views of 1 dpf embryos, which continued to exhibit *orange* coloration in the yolk and yolk extension. **(F)** Lateral view of a 5 dpf larva that appeared developmentally delayed with an uninflated swim bladder (*arrow*), cardiac edema (*white arrowhead* and *inset*, enlarged 200%), and an enlarged *orange*-colored yolk (*black arrowhead*). Scale bars = 1 mm (**A** applies to **A** and **D**; **A′** applies to **A′**, **B, B′, D′, E,** and **E′; C** applies to **C** and **F**). Hours postfertilization, hpf; days postfertilization, dpf.

The yolks of zebrafish embryos from the University of Minnesota ZCF began to exhibit an orange color, which was markedly different from normal yolks that are clear or have only a pale yellow tint (Fig. 1). This color change was observed across all genetic lines (wild type, transgenic, and mutant) in the facility and was not observed in larvae brought into the ZCF from outside facilities (data not shown). Abnormal yolk color and size were readily observable in all embryos and larvae, with greater defects evident after 3 days postfertilization (dpf). These defects included morphological deformities, behavioral abnormalities, and developmental delays. The morphological deformities observed in the embryos/larvae included cardiac edema, misshapen and enlarged yolks, and the absence of an inflated swim bladder (Fig. 1F). Behaviorally, larvae did not maintain buoyancy (sank when not actively swimming) and demonstrated a general reduction in swimming activity.

Some ZCF users also reported that larvae raised at the ZCF had anecdotally reduced rates of survival to adulthood. In addition, larval survival rates (up to 7 dpf), although not quantified, were highly variable and depended upon the incubator in which they were raised. Some incubators contained fluorescent lights running on a standard 14-h light/10-h dark schedule, while others were unlit, with embryos and larvae only receiving low-level ambient light through tinted incubator doors. Therefore, we tested the effects of lighting condition on development and mortality rates of embryos and larvae with orange-colored yolks.

**FIG. 2.**
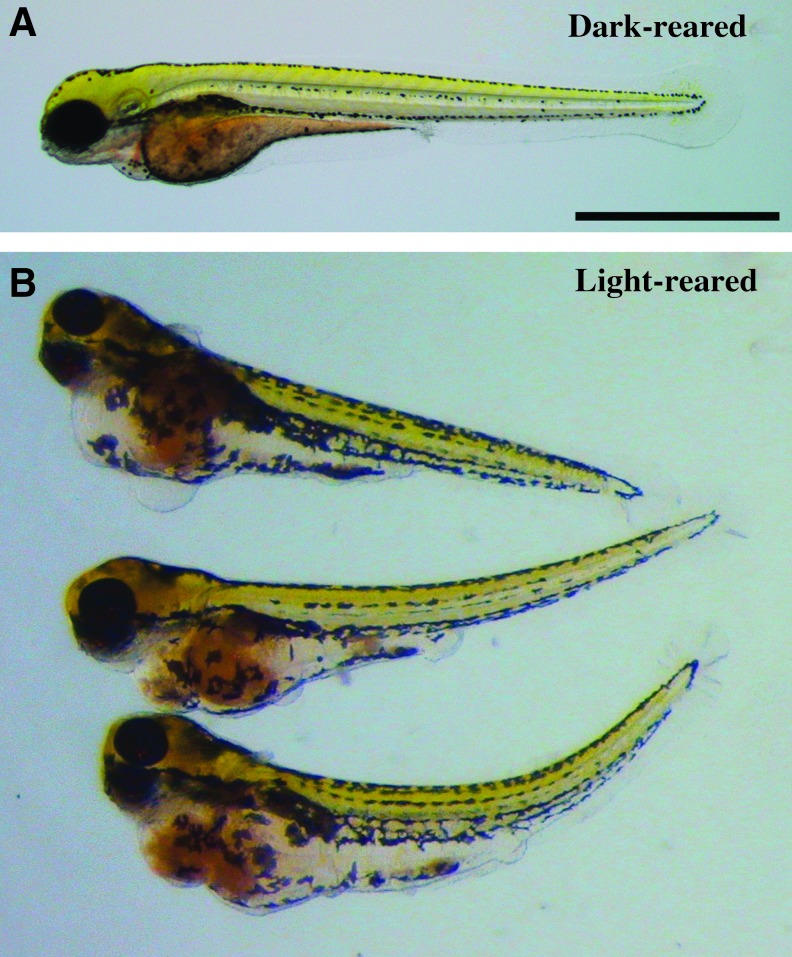
Gross morphological differences between clutch mate larvae reared in dark and light conditions. Progeny of adult zebrafish that were fed decapsulated brine shrimp cysts were raised either in complete darkness (Dark-reared) or under a normal 14-h light/10-h dark cycle (Light-reared), and larvae were compared at 5 dpf. **(A)** Dark-reared larvae were developmentally delayed and exhibited cardiac edema and enlarged *orange*-*colored* yolks, but typically maintained equilibrium and were motile. **(B)** Light-reared larvae exhibited gross morphological and developmental defects, were unable to maintain equilibrium along the transverse axis, and were mostly immotile. Scale bar = 1 mm. Days postfertilization, dpf.

Larvae reared entirely in darkness (Dark-reared) exhibited cardiac edema and were developmentally delayed, yet typically maintained equilibrium and were motile (Fig. 2A). Interestingly, larval clutch mates that were raised in normal 14-h light/10-h dark conditions (Light-reared) possessed gross morphological and developmental defects, were unable to maintain equilibrium along the transverse axis, and were largely immotile (Fig. 2B).

Mortality rates, defined as death or severe morphological defects (as shown in Fig. 2B), were quantified from 12 clutches of embryos. Between 4 and 8 h postfertilization, unfertilized embryos were removed and the remaining fertilized embryos were split into Dark-reared and Light-reared groups. By 7 dpf, 1.1% of Dark-reared larvae either had died or exhibited gross morphological defects, while 96.1% of Light-reared larvae had died or exhibited gross defects. Furthermore, the severity of symptoms appeared to be greater in larvae that had darker orange yolk coloration. Thus, orange yolk color was an indicator of poor larval health, and the severity of symptoms was exacerbated by light.

### Water quality analysis

Since heavy metal contaminants in water are known to cause developmental defects in fish,^9^ the ZCF's recirculating system water and water source were tested for the presence of heavy metals. Testing showed that arsenic, barium, and chromium were present in the system water, although below the concentrations experimentally shown to affect zebrafish reproduction and development,^10–14^ and at levels well below the maximum permitted for human consumption according to the U.S. Environmental Protection Agency National Primary Drinking Water Regulations (Table 2). These contaminants were not detected in water collected from the ZCF's reverse osmosis source; therefore, we concluded that they were introduced from an external source, potentially diet.

**Table 2.** Heavy Metal Concentrations in the Recirculating System Water at the University of Minnesota ZCF

	*Heavy metal (μg/L)*		
	*Arsenic*	*Barium*	*Chromium*
System Water	1.0	3.2	3.5
Minimum concentration shown to affect zebrafish	50^14^	80^11^	21000^13^
National primary drinking water regulations^a^	10	2000	100

^a^ U.S. Environmental Protection Agency.

### Dietary analysis

At the time that the symptoms appeared, zebrafish adults in the ZCF were fed Decaps (Brine Shrimp Direct, Ogden, UT) twice per day at ∼3.3 mg/fish/feeding and supplemented with Zebrafish Select Food (Aquaneering, San Diego, CA) thrice per week at ∼12 mg/fish/feeding. The Zebrafish Select Food was eliminated from the feeding regimen to determine if it was the cause of the observed symptoms. This diet was chosen for elimination because it was used as a supplemental feed and was therefore the simplest change to implement. However, the yolk coloration and larval health issues were still evident 2 months after its discontinuation. Thus, it was unlikely that the Zebrafish Select Food was the cause of the symptoms.

Salmonid fish produce eggs with distinctive yellow, orange, or red coloration if carotenoids are present. However, carotenoids must be exposed to salmonids through diet, as they are not synthesized internally.^15^ Once ingested, carotenoids are stored in muscle and are later transferred into ova upon sexual maturation.^16^ The emergence of orange coloration in embryonic zebrafish yolks was noted ∼2–3 weeks after the onset of feeding the adult fish in the ZCF a new lot of Decaps. Therefore, we reasoned that the change in yolk coloration from pale yellow to orange might have been due to carotenoids present in the Decaps. The ZCF obtained a sample of a previous lot of Decaps (Brine Shrimp Direct, Ogden, UT) to compare carotenoid concentrations between lots. Testing revealed that carotenoid concentrations of the new lot were lower (18 mg/kg) than in the old lot (25 mg/kg), eliminating excess carotenoids as a potential cause of the observed symptoms.

Next, to test if the presence of other potential contaminants in the Decaps contributed to the poor health and orange-colored yolks in the embryos, we acquired adult zebrafish from the University of South Carolina Aiken (USCA) zebrafish facility. While at the USCA facility, these adult fish received live brine shrimp nauplii (Artemia International, Fairview, TX), hatched in-house, in addition to a blend of dry foods: a base of Zeigler Adult Zebrafish Diet (Zeigler Bros., Gardners, PA) supplemented with Golden Pearls Fry and Coral Reef Food (Your Fish Stuff), spirulina flake food (Ocean Star International, Snowville, UT), and Thera+ A Sinking Pellets (New Life International, Homestead, FL). Once acquired and placed in the ZCF, these fish were not fed Decaps (Decap-free). Rather, they were fed exclusively GEMMA Micro feed (Skretting USA, Tooele, UT) and were exposed to the same system water as were fish native to the ZCF that had regularly been fed Decaps (Decap-fed).

Incrosses of the Decap-free adult fish resulted in healthy embryos and larvae without orange yolk coloration (Fig. 1A–C). Crosses of Decap-free adult females to Decap-fed males also produced healthy offspring without coloration in the yolks. However, offspring from crosses of the Decap-free adult males to Decap-fed females produced orange-colored yolks and exhibited all of the deformities and behavioral deficits described above. Thus, orange-colored yolks and poor larval health were only present in offspring of female fish that were raised in the ZCF and were fed a diet that included Decaps.

**FIG. 3.**
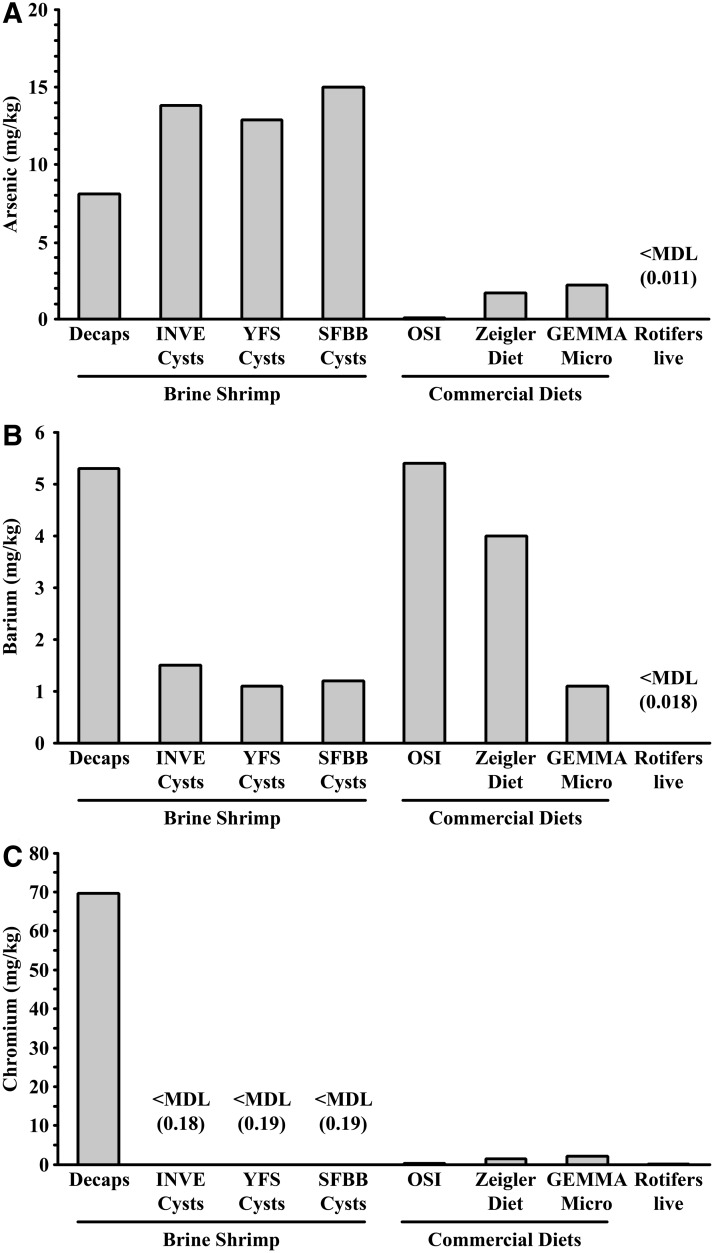
Elevated concentrations of chromium were present in decapsulated brine shrimp cysts. Comparisons of arsenic **(A)**, barium **(B)**, and chromium **(C)** levels measured in brine shrimp cysts, commercial diets, and live rotifers. Diets tested: Brine Shrimp Direct nonhatchable decapsulated brine shrimp cysts (Decaps), INVE Aquaculture Nutrition hatchable brine shrimp cysts (INVE), Your Fish Stuff hatchable brine shrimp cysts (YFS), San Francisco Bay Brand hatchable brine shrimp cysts (SFBB), Ocean Star International, Inc. artificial plankton/rotifer (OSI), Ziegler Bros., Inc. Adult Zebrafish Diet (Ziegler Diet), GEMMA Micro 300 (GEMMA Micro), *Brachionus plicatilis* L-Type Rotifer (Rotifers live). Adjusted method detection limit, MDL (MDL provided if measured value was <MDL). MDL, method detection limit.

Since the Decaps were associated with orange yolk coloration and poor larval health, and heavy metals were detected in the ZCF system water (Table 2), the Decaps were tested for the presence of heavy metals. We found that arsenic, barium, and chromium were present in the Decaps (Table 3), and although the concentration of chromium appeared to be high, there was not a frame of reference for zebrafish diets. Therefore, we also tested for heavy metals in several common zebrafish feeds, which included brine shrimp cysts (nonhatchable Decaps and hatchable cysts), commercially available diets, and live rotifers (Fig. 3).

**Table 3.** Heavy Metal Concentrations in Decapsulated Cysts, Hatchable INVE Cysts, and Hatched Nauplii

	*Heavy metal (mg/kg)*		
	*Arsenic*	*Barium*	*Chromium*
ZCF Decaps (Brine shrimp direct)	8.1	5.3	69.6
CZAR Decaps (Brine shrimp direct)	9.4	7.3	62.5
CZAR Decaps (Your fish stuff)	9.3	4.2	68.5
INVE cysts, unhatched	18.1	1.7	<0.5
INVE nauplii, hatched	2.3	<0.5	<0.5

The concentration of arsenic (Fig. 3A) was lower in the Decaps (8.1 mg/kg) than in hatchable brine shrimp cysts (12.9–15.0 mg/kg), but was greater than other commercial diets (0.1–2.2 mg/kg) and live rotifers (<adjusted MDL; 0.011 mg/kg). The concentration of barium (Fig. 3B) was greater in the Decaps (5.3 mg/kg) than in hatchable brine shrimp cysts (1.1–1.5 mg/kg) and live rotifers (<MDL; 0.018 mg/kg), but was similar to commercial diets (1.1–5.4 mg/kg). However, the concentration of chromium (Fig. 3C) was more than 30 times greater in the Decaps (69.6 mg/kg) than in hatchable brine shrimp cysts (<MDL; 0.18–0.19 mg/kg each), commercial diets (0.3–2.1 mg/kg), or live rotifers (0.04 mg/kg). These results revealed elevated concentrations of chromium in the Decaps, potentially introduced during processing, since measurable concentrations of chromium were not detected in any of the hatchable brine shrimp cysts that were tested.

### Tissue analysis

Finally, to determine if the presence of heavy metal-contaminated Decaps in the diet of the adult breeding population corresponded to elevated concentrations of chromium in embryos, heavy metal concentrations were measured and compared between a clutch of embryos obtained from Decap-fed adults (with orange-colored yolks) and a clutch of embryos obtained from Decap-free adults (with noncolored yolks). The concentration of arsenic did not exceed the MDL (0.011 mg/kg each) in either clutch. The concentration of barium was lower in the clutch collected from Decap-fed adults (<MDL; 0.019 mg/kg) than in the clutch from Decap-free adults (0.034 mg/kg). Finally, the concentration of chromium was over three times higher in the clutch of embryos collected from Decap-fed adults (0.17 mg/kg) than in the clutch from Decap-free adults (0.048 mg/kg). Altogether these data suggested that feeding chromium-contaminated Decaps to female adult zebrafish led to transmission of chromium to offspring and corresponded to orange yolk color and poor larval health and survival.

### Reclamation

Once it was determined that the Decaps were contaminated with high concentrations of the heavy metal chromium, the Decaps were immediately eliminated from the ZCF feeding regimen. All tank lids were cleaned, and floors were swept and scrubbed to remove any remnants of Decaps from the facility. The University of Minnesota Department of Health and Safety was immediately contacted to assess exposure levels of the facility staff. It was determined that human exposure due to handling was limited. However, fish exposure was of concern as ingestion is one of the primary routes of heavy metal accumulation in the body.^17,18^ Therefore, Decaps were replaced by a commercially produced pellet food (GEMMA Micro, Skretting USA, Tooele, UT). Within 3 weeks, the orange color of the yolks began to diminish, the morphological abnormalities began to subside, and larval survival began to increase in new clutches.

Clutches of embryos with orange-colored yolks, developmental delays, and behavioral defects were occasionally found in spawning tanks up to 10 months after the change in diet. Interestingly, these embryos were progeny of adult females that had not routinely spawned since the diet change. Over a period of several months, subsequent spawning sessions from those individuals resulted in a gradual return to the normal, pale yellow yolk in embryos. These results suggest that chromium accumulated in the ova of adult females that were fed the contaminated food and the effects of the heavy metal contamination could persist for several months following its elimination from the diet regimen.

**FIG. 4.**
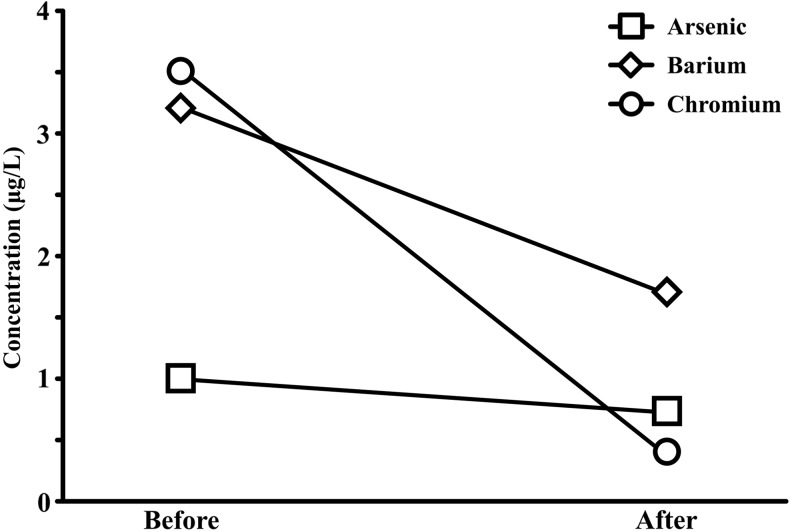
Comparison of heavy metal concentrations in the recirculating system water before (Before) and 5 months after (After) cessation of feeding with decapsulated brine shrimp cysts (Decaps). The concentrations of barium and chromium were reduced in the recirculating system water 5 months after elimination of Decaps from the feeding regimen.

Finally, to determine if the heavy metal levels in the recirculating system had changed, water samples were collected 5 months after Decaps were removed from the diet regimen. The concentrations of chromium (3.5–0.4 μg/L), barium (3.2–1.7 μg/L), and arsenic (1.0–0.7 μg/L) were reduced in the recirculating system water 5 months after elimination of Decaps from the feeding regimen (Fig. 4).

### The University of Utah CZAR

Similar symptoms, such as discoloration of embryos and cardiac edema, were observed during the same time period at the CZAR. The CZAR fed adult fish a combination of Decaps (Your Fish Stuff), hatched nauplii (INVE, Salt Lake City, UT), and Tropical Flakes (Drs. Foster and Smith, Rhinelander, WI). The CZAR submitted INVE cysts and 24 h-old (first instar stage) INVE nauplii for heavy metal testing. An eightfold decrease in arsenic concentration was measured between the cysts (18.1 mg/kg) and their hatched nauplii (2.3 mg/kg; Table 3). This indicated that the high concentration of arsenic present in the cysts was reduced following hatching of the nauplii. The CZAR also tested two brands of Decaps, which revealed concentrations of arsenic, barium, and chromium that corresponded to those at the ZCF (Table 3). Similarly, symptoms in embryos produced at the CZAR diminished approximately 1 month after removing the Decaps from the diet regimen.

## Discussion

The morphological deformities, orange yolk coloration, and increased mortality are presumed to be a result of feeding adult zebrafish Decaps that were contaminated with heavy metals, specifically chromium. Although some aquatic pathogens are associated with altered behavior in larval zebrafish,^19^ there is no evidence in the literature that known pathogens are capable of producing the symptoms observed in larvae at the ZCF and the CZAR, particularly the orange yolk coloration. After elevated concentrations of arsenic, barium, and chromium were detected in the ZCF recirculating system water (Fig. 4 and Table 2), feeds were tested as a potential exogenous source of heavy metal contamination. Decaps, the primary component of the diet regimen at the ZCF, contained elevated concentrations of chromium compared to the other feeds tested (Fig. 3). Upon removing the Decaps from the diet regimen, yolk color quickly returned to normal and health slowly improved.

Arsenic concentrations for all types of brine shrimp cysts (hatchable and Decaps) were over four times higher than commercial diets and rotifers, ranging from 8 to 15 mg/kg (Fig. 3A). Brine shrimp cysts are often harvested from the wild and, thus, may be susceptible to bioaccumulation of environmental contaminants such as arsenic.^20^ The European Union Directive on undesirable substances in animal feeds (2002/32/EC) states that the arsenic concentration in complete feeds for fish should not exceed 10 mg/kg,^21^ lower than concentrations measured in all three brands of hatchable brine shrimp cysts tested (Fig. 3A). Some arsenic compounds are lethal in mice at doses as low as 0.9 g/kg,^22^ above the 8 mg/kg found in the Decaps. Lake whitefish (*Coregonus clupeaformis*) and rainbow trout exhibit decreased growth and food consumption when fed arsenic at 100 mg/kg and 26 mg/kg, respectively.^23,24^ Chronic inflammation of the gallbladder in rainbow trout was evident when they were fed arsenic in amounts as low as 33 mg/kg.^25^ Additional research on specific arsenic compounds and their effects on zebrafish development is needed to determine what levels are acceptable for zebrafish culture.

The high concentrations of arsenic in hatchable cysts were of concern since newly hatched nauplii, which are derived from hatchable cysts, are a primary food source for many zebrafish facilities. With this in mind, the CZAR tested arsenic in both the INVE hatchable cysts and the nauplii derived from the INVE hatchable cysts. A higher concentration of arsenic was present in the unhatched cysts (18.1 mg/kg) than in hatched nauplii (2.3 mg/kg; Table 3), which suggested that arsenic was either excreted during hatching and early development of the nauplii or that it was concentrated in the chorion. Additional data on the arsenic levels in hatched nauplii from other suppliers are needed to confirm this finding.

Elevated barium is not a likely cause of the symptoms observed in the ZCF and the CZAR. First, the concentration of barium in Decaps was similar to other common zebrafish diets (Fig. 3B). Second, the symptoms described above were alleviated in progeny when adult fish in the facility were switched to other feed sources, which contained similar concentrations of barium (Fig. 3B). Finally, the concentration of barium detected in Decaps (5.3 mg/kg at ZCF and 4.2 mg/kg at the CZAR) was much lower than what was found to be toxic to mice (4000 mg/kg).^26^

Chromium is carcinogenic, can disturb embryo development, and causes mutations in numerous organisms.^27^ Furthermore, chromium is neurotoxic, interferes with cellular metabolic activity, and inhibits glutathione S-transferase activity in larval zebrafish when exposed to high levels in the water.^13^ Research focused on chromium oral toxicity in fish is quite limited. Dietary hexavalent chromium above 60 mg/kg perturbs cholinergic signaling in juvenile rock fish (*Sebastes schlegelii*),^28^ while 3.2 mg/kg has little effect on Japanese medaka.^29^ More is known about dietary chromium toxicity in rodents. Incidence of adenoma and carcinoma in mice increased when 30 mg/L chromium was consumed through their drinking water.^30^ Depending on the chromium compound, oral acute toxicity for rats ranges between 50 and 12,000 mg/kg.^31^ The total chromium concentration found in the Decaps (69.6 mg/kg) was within the range of these studies, yet further research on specific chromium compounds is needed to determine their effects on zebrafish development and transmission to offspring.

Tissue analysis of ZCF embryos revealed that embryos with orange-colored yolks that were obtained from Decap-fed adults contained over three times more chromium than embryos with noncolored yolks obtained from Decap-free adults. The correlation between chromium and yolk coloration could be explained by the fact that chromium compounds can be intensely colored and are used as an industrial dye to create colors such as yellow, red, green, and orange.^32^ We hypothesize that the high concentration of chromium found in zebrafish embryos resulted from adult female consumption of chromium-contaminated food, which then accumulated in the ova. Consistent with this hypothesis, maternal transfer of chromium was reported in Japanese medaka (*Oryzias latipes*) and is an important pathway for chromium deposition into offspring.^33^

The severity of symptoms observed at the ZCF appeared to be greater in embryos and larvae that had darker orange yolk coloration, and symptoms were exacerbated by exposure to light (Fig. 2). Chromium significantly increases the incidence of skin tumors in hairless mice that are exposed to ultraviolet radiation,^34^ and it has been suggested that synergism occurs between the two.^35^ This is consistent with our findings and presents an intriguing avenue for future work, particularly using transparent zebrafish larvae or pigment-free mutant adults, such as *casper*.^36^

Chromium concentrations in Decaps were over 30 times higher than all other feeds analyzed (Fig. 3C) and are thought to be the cause of the symptoms observed at the ZCF and the CZAR. The high concentrations of chromium in the Decaps (69.5 mg/kg) were not likely due to environmental bioaccumulation, as other brine shrimp cysts contained <0.19 mg/kg chromium, and the highest level reported in crustaceans harvested from the wild was 0.6 mg/kg.^27^ The chromium concentration in the Decaps was at least 100 times higher than that measured in hatchable brine shrimp cysts (Fig. 3) and what has been reported in the wild, which suggests that contamination occurred after harvesting. However, we were unable to test the cysts before decapsulation and therefore could not determine if chromium contamination was the result of processing and distribution or harvesting of contaminated cysts.

Most zebrafish facilities use a combination of feeds, and the feeds used within a facility often change due to feed availability and the process of optimizing diets to improve fish health and reproduction. Feed quality is not well regulated, and the standards of suppliers are quite variable. The incidents that occurred at the ZCF and the CZAR provide an example of how the implementation of a standardized diet and feed quality control may benefit zebrafish health, thereby reducing the likelihood of introducing contaminants that may affect the rigor and reproducibility of research.
